# Identification and characterization of the three members of the CLC family of anion transport proteins in *Trypanosoma brucei*

**DOI:** 10.1371/journal.pone.0188219

**Published:** 2017-12-15

**Authors:** Michael E. Steinmann, Remo S. Schmidt, Juan P. Macêdo, Christina Kunz Renggli, Peter Bütikofer, Doris Rentsch, Pascal Mäser, Erwin Sigel

**Affiliations:** 1 Institute of Biochemistry and Molecular Medicine, University of Bern, Bern, Switzerland; 2 Swiss Tropical and Public Health Institute, Basel, Switzerland; 3 University of Basel, Basel, Switzerland; 4 Institute of Plant Sciences, University of Bern, Bern, Switzerland; Albany Medical College, UNITED STATES

## Abstract

CLC type anion transport proteins are homo-dimeric or hetero-dimeric with an integrated transport function in each subunit. We have identified and partially characterized three members of this family named TbVCL1, TbVCL2 and TbVCL3 in *Trypanosoma brucei*. Among the human CLC family members, the *T*. *brucei* proteins display highest similarity to CLC-6 and CLC-7. TbVCL1, but not TbVCL2 and TbVCL3 is able to complement growth of a CLC-deficient *Saccharomyces cerevisiae* mutant. All TbVCL-HA fusion proteins localize intracellulary in procyclic form trypanosomes. TbVCL1 localizes close to the Golgi apparatus and TbVCL2 and TbVCL3 to the endoplasmic reticulum. Upon expression in Xenopus oocytes, all three proteins induce similar outward rectifying chloride ion currents. Currents are sensitive to low concentrations of DIDS, insensitive to the pH in the range 5.4 to 8.4 and larger in nitrate than in chloride medium.

## Introduction

*Trypanosoma brucei ssp*. are the causative agents of human African trypanosomiasis (HAT), also called sleeping sickness, and of a related disease in livestock, called nagana. Untreated sleeping sickness is fatal. The parasites are transmitted by the tsetse fly (*Glossina spp*.). During their life cycle, the parasites must tightly regulate their internal ion composition to survive and proliferate in different environments such as the tsetse fly gut, salivary glands, or the human blood. Until recently, transporters or channels selective for inorganic ions have attracted little attention. Research in *T*. *brucei* transport systems has so far mainly concentrated on transporters of organic nutrients such as amino acids, sugars, nucleosides, nucleobases and metabolites [1–5].

Anion transport proteins that are members of the CLC gene family are found in all three domains of life. The first CLC member was identified in *Torpedo marmorata* and described as voltage-gated chloride channel [6]. Later it became apparent that some CLCs function as electrogenic Cl^-^/H^+^-exchangers [7]. CLCs are dimers and have two ion translocation pathways, one in each subunit [8,9]. Formation of homodimers as well as heterodimers has been observed in heterologous expression systems [8]. Some mammalian CLC proteins also incorporate the β-subunits Barttin, Ostm1 or GlialCam [10–12]. In the human genome there are nine isoforms, termed CLC-1 to CLC-7, CLC-Ka and CLC-Kb. Human CLC-1 is located in the skeletal muscle plasma membrane and involved in the stabilization of the membrane potential. CLC-2, CLC-Ka and CLC-Kb are implied in transepithelial transport. CLC-3 to CLC-7 have intracellular functions in synaptic vesicles and/or endosomes and lysosomes (reviewed in [13]).

In trypanosomes, no chloride transporters have yet been described. Here, we show that three proteins conveying this function are present in *T*. *brucei* procyclic forms. After expression *Xenopus* oocytes the resulting currents were functionally characterized. All three proteins are reversibly inhibited by 4,4-diisothiocyanostilbene-2,2-disulphonate (DIDS) and transport different anions such as chloride, bromide, iodide and nitrate. In *T*. *brucei* procyclic forms all three proteins localize intracellularly.

## Materials and methods

### Materials

Unless otherwise noted, all reagents were obtained from Sigma-Aldrich (St. Louis, MO, USA).

### Phylogenetic analysis of TbVCLs and related proteins

Protein sequences of CLC proteins in humans, *Arabidopsis thaliana*, *Saccharomyces cerevisiae* and three members of the tri-trypanosomatid group (*Trypanosoma brucei*, *T*. *cruzi* and *Leishmania major*) were aligned with MUSCLE [14]. Loci for *Arabidopsis* genes from AtClCa to AtClCg are as follows; AT5G40890, AT3G27170, AT5G49890, AT5G26240, AT4G35440, AT1G55620, AT5G33280. Following the alignment, we grew a minimum evolution tree. The complete analysis was carried out in MEGA6 [15]. Visualization of the tree was done in Dendroscope [16].

### Identification of CLC proteins in *Xenopus laevis*

To obtain all CLC family proteins in *Xenopus laevis*, we scanned all peptides predicted from gene models version 1.8.3.2 of the assembled *Xenopus laevis* genome version 9.1, acquired from http://www.xenbase.org/ as follows. As query, we used the Pfam family PF00654 HMM logo, obtained from http://pfam.xfam.org/, and searched with HMMsearch version 3.1b2. The search produced 18 peptides, whereof 15 had E-values between 2.5E-53 and 9.1E-110 for the full sequence. The remaining three hits were of low confidence (E-values between 0.0063 and 0.015). In the 15 hits with a low E-value, we found the genes clcn1 through clcn7, clcnkb, and three additional genes. The genes clcn1 through clcn4 were found on both short and long chromosomes; clcn5 through clcn7 and clcnkb were only found on a long chromosome. In addition, we found loc100488828 on a long and loc100487225 on a long and a short chromosome, also bearing the CLC family member motif.

### Sequence alignment

Protein sequences for the described human CLC proteins, the yeast protein GEF1, the *E*. *coli* protein ClC-ec1, and the three *T*. *brucei* proteins were aligned using MUSCLE. Alignment and graphical representation was carried out in CLC sequence viewer 7.7.

### Expression in *Xenopus* oocytes

Animal experiments were carried out in strict accordance to the Swiss ethical guidelines, and have been approved by the Kantonstierarzt of the Canton Bern, Kantonaler Veterinärdienst Bern (BE85/15). Surgery of *Xenopus laevis* to obtain the oocytes was done under anesthesia with 0.2% tricaine solution (ethyl aminobenzoate, MS222) and analgesic treatment with 25mg/kg Flunixin-Meglumin. *Xenopus laevis* oocytes were prepared, injected and defollicated as described previously [17]. Polyadenylated cRNAs coding for Tb927.9.8540 (TbVCL1), Tb927.10.11680 (TbVCL2) and Tb927.11.16690 (TbVCL3) were prepared *in vitro* with the mMESSAGE mMACHINE kit (Ambion, Austin, TX, USA). Oocytes were injected with 50 nl of solution containing cRNA coding for one of the genes (0.4 μg/μl) or water and then incubated in modified Barth’s solution (10 mM HEPES, pH 7.5, 88 mM NaCl, 1 mM KCl, 2.4 mM NaHCO_3_, 0.82 mM MgSO_4_, 0.34 mM Ca(NO_3_)_2_, 0.41 mM CaCl_2_, 100 units/ml penicillin, 100 μg/ml streptomycin) at 18°C for 3 days before measurements.

### Functional characterization in *Xenopus* oocytes

Electrophysiological experiments were performed using an Oocyte Clamp OC-725 (Warner Instrument Corp., Hamden, USA) two-electrode voltage clamp amplifier. Currents were digitized at 5 kHz with MacLab/200 (AD Instruments, Spechbach, Germany).

The holding potential was -40 mV. With a frequency of 1 Hz discrete voltage steps of 300 ms duration to potentials ranging from -120 mV to +80 mV in steps of 10 mV were applied. The perfusion medium contained 90 mM NaCl, 1 mM KCl, 1 mM MgCl_2_, 1 mM CaCl_2_ and 5 mM Na-HEPES (pH 7.4) (chloride medium). The perfusion solution (6 ml/min) was applied through a glass capillary with an inner diameter of 1.35 mm, the mouth of which was placed about 0.5 mm from the surface of the oocyte. The ground electrode contained 3% agar in 3M KCl. Other media used for the functional characterization were the following: gluconate medium where gluconate was used as the major anion containing 90 mM Na-gluconate, 1 mM KCl, 1 mM MgCl_2_, 1 mM CaCl_2_ and 5 mM Na-HEPES (pH 7.4). To determine the anion selectivity, 90 mM NaCl in the chloride medium were replaced by either 90 mM NaBr (bromide medium), 90 mM NaI (iodide medium) or 90 mM NaNO_3_ (nitrate medium).

### Yeast transformation and growth upon iron deprivation

TbVCL1 and TbVCL2 were cloned into the yeast expression vector pDR195 [18] between restriction sites *Xho*I/*Not*I and *Not*I/*Bam*HI, respectively. TbVCL3 was cloned into pDR196 between restriction sites *Spe*I and *Eco*RI. *S*. *cerevisiae* Δ*gef1*::*Kan*^*R*^ mutant strain Y16838 (BY4742; *Matα*, *his3*Δ*1*, *leu2*Δ*0*, *lys2*Δ*0*, *ura3*Δ*0*, *YJR040w*::*kanMX4*; EUROSCARF), was transformed as described [19] and plated on SD medium without uracil. For the iron deprivation assay, individual colonies were transferred to liquid SC medium (containing all amino acids, but lacking uracil) and grown to an optical density (O.D._600_) of ~0.6. Cells were centrifuged, adjusted to an O.D._600_ of 0.1 in water, washed once and resuspended in SC medium to an O.D._600_ of approximately 0.02. A serial dilution (1:2) of bathophenanthrolinedisulfonic acid (BPS) (starting with 120 μM final concentration) was prepared in a 96-well plate with round bottom wells, containing 100 μL per well. One hundred microliters of resuspended cells were added to each well in duplicates and incubated at 28°C with constant shaking. As control yeast were grown in the absence of BPS. At defined time points, the cell suspensions were mixed using a multichannel pipette and the O.D._600_ was measured using a microplate reader (Multiscan Ascent, ThermoScientific).

### Quantification of TbVCL-transcripts in yeast

For total RNA extraction from *S*. *cerevisiae*, cells were pre-incubated at 30°C for 30 min in 200 μL lyticase lysis solution (1 M sorbitol, 0.1 mM EDTA, 0.1% beta-mercaptoethanol and ~100 U lyticase). One hundred and fifty microliters of Promega SV RNA lysis solution (Promega, Madison, USA) was added to each sample and RNA isolation was continued following the manufacturer’s instructions. RNA samples were treated with DNase I (Roche, Basel, Switzerland) for 25 min at 37°C, followed by phenol/chloroform extraction and ethanol precipitation. DNase I-treated RNA (0.5 μg) was used for cDNA synthesis using Takara PrimeScript reverse transcriptase (Takara, Shiga, Japan). Quantitative RT-PCR was performed using a LightCycler 480 System (Roche). The reaction mixtures consisted of 1x SYBR green premix, *Ex taq* (RR420L, Takara) and 0.2 μM forward and reverse primers. Two reference genes [20] were amplified using primers ALG9_F, 5’- CACGGATAGTGGCTTTGGTGAACAATTAC-3’ and ALG9_R, 5’-TATGATTATCTGGCAGCAGGAAAGAACTTGGG-3’; and TAF10_F, 5’- ATATTCCAGGATCAGGTCTTCCGTAGC-3’ and TAF10_R, 5’- GTAGTCTTCTCATTCTGTTGATGTTGTTGTTG-3’. For quantification of TbVCLs-transcripts the same primers were used as for the analysis of parasites (described below).

### Stable transfection of trypanosomes and selection of clones

*T*. *brucei* 29–13 procyclic forms [21] were cultured to mid-log phase (0.5–0.8 × 10^7^ cells/ml) in SDM-79 (BioConcept, Allschwil, Switzerland) supplemented with 10% (v/v) fetal bovine serum (FBS, LuBioScience GmbH, Lucerne, Switzerland) at 27°C. Parasites (4–5 × 10^7^) were harvested by centrifugation at 1250 × *g* for 10 min, washed once in transfection buffer (132 mM NaCl, 8 mM KCl, 8 mM Na_2_HPO_4_, 1.5 mM KH_2_PO_4_, 0.5 mM magnesium acetate, 0.09 mM calcium acetate, pH 7.0), resuspended in 450 μl of the same buffer, and mixed with 15 μg of linearized plasmid. Electroporation was performed with a BTX Electroporation 600 System (Axon Lab, Baden, Switzerland) with one pulse (1.5 kV charging voltage, 2.5 kV resistance, 25 mF capacitance timing, and 186 ohm resistance timing) using a 0.2-cm pulse cuvette (Bio-Rad Laboratories AG, Cressier, Switzerland). Electroporated cells were immediately inoculated in 10 ml SDM-79 containing 10% heat-inactivated FBS. Clones were obtained by limited dilution in 24-well plates in SDM-79, containing 20% conditioned medium, in the presence of 2 μg/ml puromycin (Invitrogen, Carlsbad, CA, USA) for selection. Antibiotic-resistant clones were tested for the presence of the introduced constructs by PCR. RNA interference and expression of HA-tagged constructs was induced by addition of 1 μg/ml tetracycline to parasite cultures.

### RNAi-mediated gene silencing

Expression of TbVCL1, TbVCL2 and TbVCL3 was down-regulated by RNAi using a stem loop construct containing a puromycin resistance gene. RNAit, a prediction algorithm designed to prevent potential cross-talk and hence off-target effects [22], was employed to select the gene sequences used for the RNAi-constructs. For TbVCL1 the 487bp fragment spanning nucleotides 1725–2212 was amplified using forward primer ATCGGAATCTAAGCTTGGATCCTGGTCTGTTTGCGCTGATC and reverse primer ATCGGAATCTTCTAGACTCGAGGTGACCAAGCCCACAAACTT. For TbVCL2 a 427bp fragment spanning nucleotides 1436–1863 was amplified using forward primer ATCGGAATCTAAGCTTGGATCCTGACGTGTCGGTCATATCGT and reverse primer ATCGGAATCTTCTAGACTCGAGTTGTGCCACCCACAAAACTA. For TbVCL3 a 354bp fragment spanning nucleotides 403–757 was amplified with forward primer ATCGGAATCTAAGCTTGGATCCTATCTTGAGCGCCGGAAG and reverse primer ATCGGAATCTTCTAGACTCGAGACAGGAACACCGGCAGATAC. The amplified fragments were cloned into vector pALC14 (modified pZJM, based on pLew100, which targets insertion to ribosomal RNA genes [21,23,24]) as described previously [25], allowing for tetracycline-inducible expression of a hairpin RNA under an rRNA promoter. For double RNAi knockdown, the insert from pALC14 targeting TbVCL3 was cloned into pMS14v5 [26], a derivative of pLew100v5, allowing selection by 2.5 μg/ml phleomycin (InvivoGen, San Diego, CA, USA) and expression of the stem-loop construct under the control of an rRNA promoter upon addition of tetracyclin to the culture medium. Before transfection of *T*. *brucei* cells, plasmid DNA was linearized with *Not*I followed by a phenol/chloroform-extraction and subsequent precipitation.

Transfection for double-knockdown of TbVCL2 and TbVCL3 mRNA was carried out by electroporating 4 × 10^7^ parasites previously transfected with the pALC14-TbVCL2-RNAi construct with 10 μg pMS14v5-TbVCL3-RNAi construct as described before [27], using program X-014 on an Amaxa Nucleofector 2b (Lonza, Basel, Switzerland).

### RNA isolation and qRT-PCR

Induced and non-induced RNAi cultures were harvested 48 h post induction and total RNA was extracted according to the manufacturers’ protocol using the Promega SV total RNA extraction Kit (Promega Corporation, Madison, WI, USA) or the RNeasy Mini Kit (Qiagen, Hilden, Germany) using the on-column DNase treatment. RNA concentration and purity were measured with a spectrophotometer (Thermo Scientific NanoDrop™ 1000, USA) and 10 μg total RNA from each time point were subjected to a second DNaseI treatment (Roche, Basel, Switzerland) when isolated with the Promega SV kit. Afterwards, the RNA was purified by a phenol/chloroform extraction followed by precipitation. RNA concentration was again determined with a spectrophotometer and 2 μg total RNA were used for reverse transcription with random primers using the High Capacity cDNA Reverse Transcription Kit (Applied Biosystems, Life Technologies Ltd., Paisley, UK) or 500 ng total RNA were used in combination with the Superscript III Reverse Transcriptase (Thermo Fisher, Waltham, MA). The resulting cDNA was diluted to the desired concentrations and analyzed by quantitative PCR using the Fast SYBR Green Kit (Applied Biosystems) according to the manufacturer’s instruction. As validated internal control the expression level of Tb927.11.10190 mRNA was measured in parallel [28]. The specific primers for the target were designed with the Primer3Plus-tool [29]. For TbVCL1 forward and reverse primer GATGCCTGTTTTCCACCAGT and CGTCTACAACCACCATGTGC were used to amplify a 138 bp long product at position 2512–2650 of the gene. For TbVCL2 forward and reverse primer ACCGTACCGGGTATTCATCA and AGCGCACCACAAATAAAACC were used to amplify a 141 bp long fragment at position 974–1115 of the gene. For TbVCL3 forward and reverse primer CTGCGGATCTTACCCACATT and TCAGGTCAAGCAGTGAGTGG were used to amplify a 104 bp long product at position 1802–1906 of the gene. For Tb927.11.10190 we used the primers described before [28]. Each sample was analyzed in triplicates and the average threshold cycle (Ct) was calculated for each sample. The relative expression levels of the target genes were determined by the 2^-∆∆CT^ method [30].

### Immunofluorescence and microscopy

To generate cell lines overexpressing HA-tagged versions of TbVCL1, TbVCL2 and TbVCL3 we used the pALC14 vector as described before [5]. For all three targets C-terminal as well as N-terminal constructs were prepared. All constructs were linearized with *Not*I, extracted with phenol/chloroform and precipitated prior to transfection. For immunolocalization, procyclic form trypanosomes were cultured in presence of 1 μg/ml tetracycline for 24 h to induce expression of the tagged proteins. Parasites were harvested by centrifugation at 800 x *g* for 10 min, washed with cold phosphate-buffered saline (135 mM NaCl, 1.3 mM KCl, 3.2 mM Na_2_HPO_4_, 0.5 mM KH_2_PO_4_, pH 7.4) and then spread on Superfrost Plus Microscope Slides (Thermo Scientific, Braunschweig, Germany). Preparation of the slides for microscopy was done as described earlier [31].

The following primary antibodies were used; mouse monoclonal anti-HA (Covance, Princeton, NJ, USA), rabbit anti-TbGRASP (kindly provided by G. Warren, University of Vienna), rabbit anti-TbCatL and rabbit anti-BiP (both a kind gift of J.D. Bangs, University of Buffalo) at dilutions of 1:250, 1:1000, 1:500 and 1:1000, respectively. The corresponding secondary fluorophore-conjugated goat anti-mouse Alexa Fluor 488 or goat anti-rabbit Alexa Fluor 594 (Invitrogen) antibodies were added at dilutions of 1:1000. Mitochondrial staining was done by incubation of the parasites for 20 min with 0.5 μM MitoTracker Red CM-H_2_XRos (Invitrogen) and subsequent washing prior to spreading the cells on the microscope slides.

Fluorescence microscopy was performed on a Leica DMI6000 B microscope (Leica Microsystems). Deconvolution and analysis of the images was done with the Leica Application Suite X software provided by the manufacturer. Separation of Golgi and HA signals in HA-TbVCL1 cells was measured in Icy [32]. To determine numbers of kinetoplasts, Golgi apparatuses and Tb-VCL1 signals, a custom Icy protocol was used to minimize observer bias by generating thresholded one-layer images. Numbers of signals were then determined by counting manually.

## Results

### Sequence analysis of TbVCL1, TbVCL2 and TbVCL3

**Fig 1:**
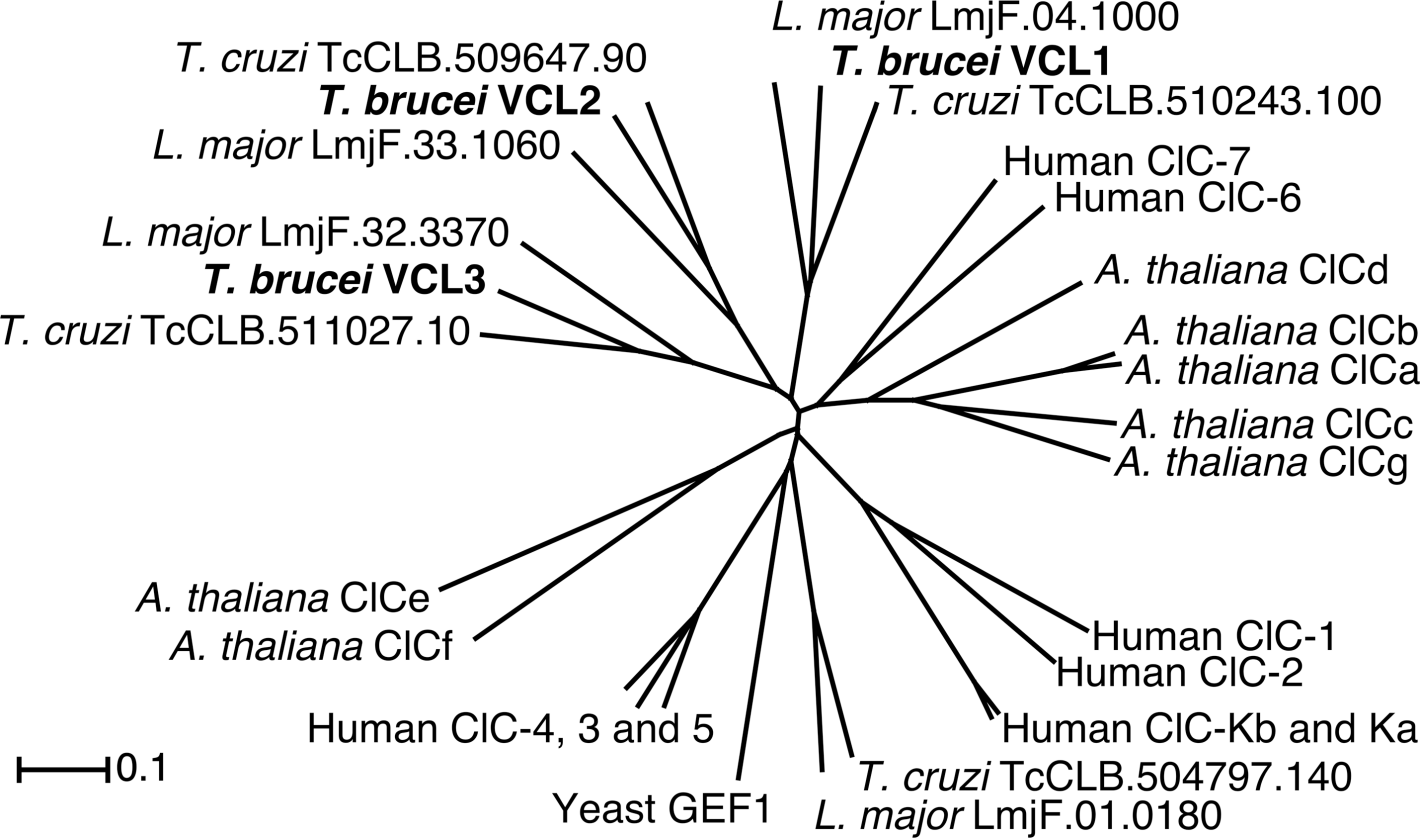
Radial phylogram of TbVCLs and related proteins. Protein sequences of TbVCLs were aligned to related proteins from other kinetoplastids, human, *S*. *cerevisiae* and *A*. *thaliana* by using MUSCLE [14]. The resulting alignment was used to grow a minimum evolution tree. Scale bar indicates nucleotide substitutions per site.

An *in silico* screen of the predicted *T*. *brucei* proteome (v4.0 from ftp.sanger.ac.uk) for ion channels using the hmmer-3.0b3 program [33] identified three genes scoring positive for the Voltage_CLC family profile (PF00654). The protein products of the identified sequences Tb927.9.8540, Tb927.10.11680 and Tb927.11.16690 were named TbVCL1 (**V**oltage-gated **C**hloride channel **L**ike), TbVCL2 and TbVCL3, respectively. Amino acid identity between the members was 24% to 27% as determined with a global alignment [34]. The orthologs of TbVCL1, TbVCL2 and TbVCL3 in *T*. *cruzi* and *L*. *major* share 54–61% and 42–49% amino acid identity, respectively. In human proteins highest similarity is found for CLC-6 (AAI17421.1) and CLC-7 (AAF34711.1) with 26–27% for TbVCL1 and TbVCL3 and 21–22% for TbVCL2. All three *T*. *brucei* VCLs contain CBS domains (cystathionine-β-synthase; PF00571) in their C-terminal regions that have been shown to be involved in the functional regulation of certain members of the CLC family [35]. Fig 1 shows a radial phylogram of the newly described trypanosome proteins, TbVCL1, TbVCL2 and TbVCL3, predicted CLC proteins in related kinetoplasts, and known transporters from *S*. *cerevisiae*, *A*. *thaliana* and humans (for bootstrap values see S1 Fig).

S2 Fig shows a sequence alignment of the described human CLC proteins, the yeast protein GEF1, the *E*. *coli* protein ClC-ec1, and the three *T*. *brucei* proteins. The selectivity filter of CLC proteins is made up by a serine that is implicated in ion selectivity, a so-called gating glutamate and a tyrosine residue. Sequence alignment with known CLC proteins indicates that the amino acid residues of the selectivity filters of TbVCL1, TbVCL2 and TbVCL3 differ from the consensus. Only TbVCL3 contains the corresponding serine, TbVCL1 and TbVCL3 contain the gating glutamate, the proton glutamate and the corresponding tyrosine residue. Thus, the protein expressing best in Xenopus oocytes (see below), TbVCL2, does not contain any of the consensus residues.

### Functional characterization by expression in a *S*. *cerevisiae gef1∆* mutant

**Fig 2:**
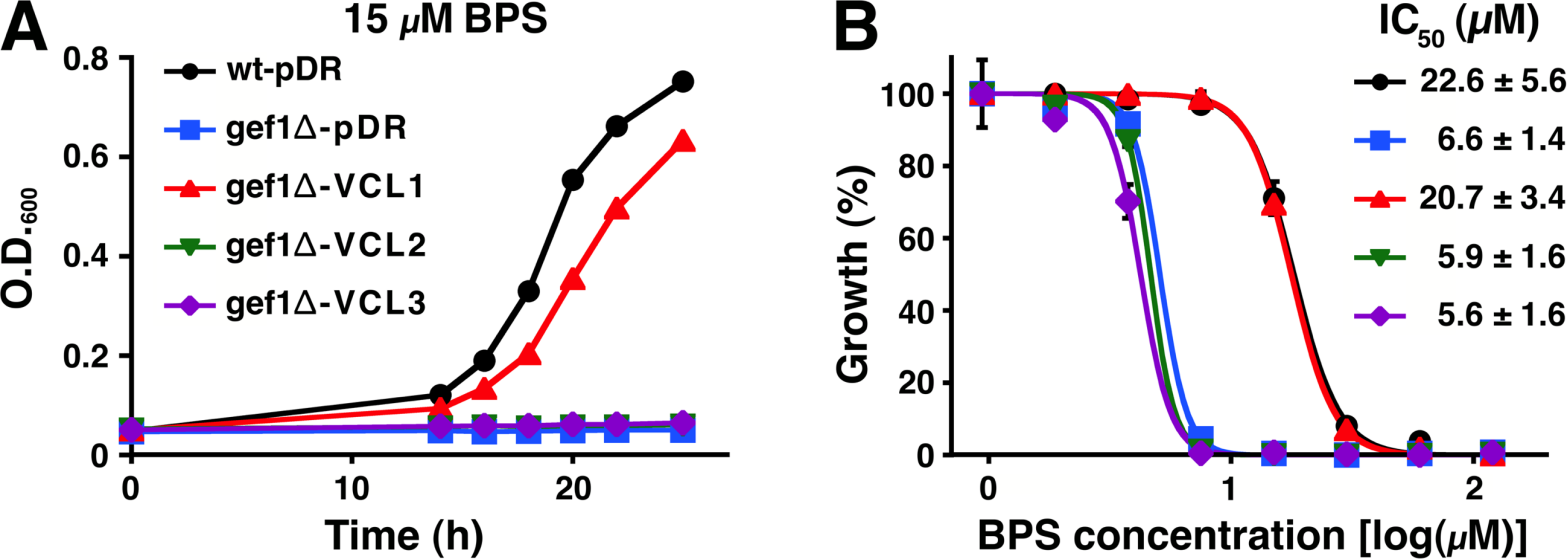
Sensitivity of *gef1∆* mutant *S*. *cerevisiae* expressing TbVCLs to iron deprivation. *Gef1∆* mutant (Y16838) transformed with vector (pDR), TbVCL1, TbVCL2 or TbVCL3 were grown in complete SC medium in the presence of 15 μM (A) or various concentrations (B) of the iron chelator bathophenanthrolinedisulfonic acid (BPS) for 25 h. Vector-transformed parental strain (BY4742) was used as a control. A) Time-dependent growth of transformants. B) Growth after 25 h incubation in the presence of various concentrations of BPS. Data points are normalized as follows: optical density (O.D._600_) values of two replicates of a representative experiment in which the maximal growth (O.D._600_ ~0.7) was adjusted to 100% and minimal growth (O.D._600_ ~0.05) to 0%. Half inhibitory concentrations (IC_50_) values were calculated from three independent experiments ± SD.

To obtain evidence for functional chloride transport. TbVCL1, TbVCL2 and TbVCL3 were individually expressed under control of the ATPase (*PMA1*) promoter in an *S*. *cerevisiae gef1∆* mutant (strain Y16838). Deletion of the *GEF1* gene, which codes for a yeast Cl^-^/H^+^-exchanger belonging to the CLC family, causes various phenotypes, but sensitivity to iron deprivation can easily be measured [36–38]. To determine whether the *T*. *brucei* VCLs were able to complement the mutant phenotype, we cultivated the cells in the presence of increasing concentrations of the iron chelator bathophenanthrolinedisulfonic acid (BPS). Expression of TbVCL1 led to almost full recovery of growth in the presence of 15 μM BPS compared to the corresponding wild-type strain (BY4742) transformed with the vector pDR (Fig 2A). Growth rescue was not observed in the *gef1∆* mutant expressing TbVCL2 or TbVCL3 at the same BPS concentration (Fig 2A). Furthermore we found that for TbVCL1 the BPS concentration of half maximal growth inhibition (IC_50_) was in the same range as for the wild-type control, while TbVCL2- and TbVCL3-expressing *gef1*∆ cells as well as the *gef1*∆ mutant transformed with the vector showed a 3- to 4-fold higher sensitivity to BPS (Fig 2B). Expression of the TbVCLs was confirmed at the RNA-level by qRT-PCR (S3 Fig).

### Electrophysiological characterization of the currents induced by TbVCL1, TbVCL2 and TbVCL3

As the yeast complementation experiments failed to show functional evidence for TbVCL2 and TbVCL3, we tried to functionally express the proteins in *Xenopus laevis* oocytes. The first indication of individual functional expression of TbVCL1, TbVCL2 and TbVCL3 was the increased outward currents of oocytes injected with cRNA compared to the water-injected control oocytes (n = 23). Increased outward currents were never detected in water-injected control oocytes or in oocytes expressing the *T*. *brucei myo*-inositol transporter [5] or the *T*. *brucei* AAT6 amino acid transporter [1]. For reasons we can only speculate about, microinjection of cRNA of TbVCL1, TbVCL2 and TbVCL3 either resulted in no extra current beyond endogenous conductances or large μA sized currents. Possibly, because transport proteins are localized in intracellular organelles in *T*. *brucei* (see below), the observed currents are due to a spillover of the transport function to the oocyte plasma membrane. It is not clear why this spillover follows an all-or-none logic. Large current signals were detected in 52% of injected oocytes for TbVCL1 (n = 29), 97% for TbVCL2 (n = 29) and 39% for TbVCL3 (n = 26). It should be noted that we can not exclude the possibility that the trypanosome proteins combine with one of the 18 CLC-like proteins found in *Xenopus laevis* (see methods for identification). In the following, characteristics found after expression of TbVCL2 are described in detail. TbVCL1, TbVCL3 or a combined expression of all the three proteins resulted in similar currents. We only analyzed data from oocytes displaying large (μA) currents.

**Fig 3:**
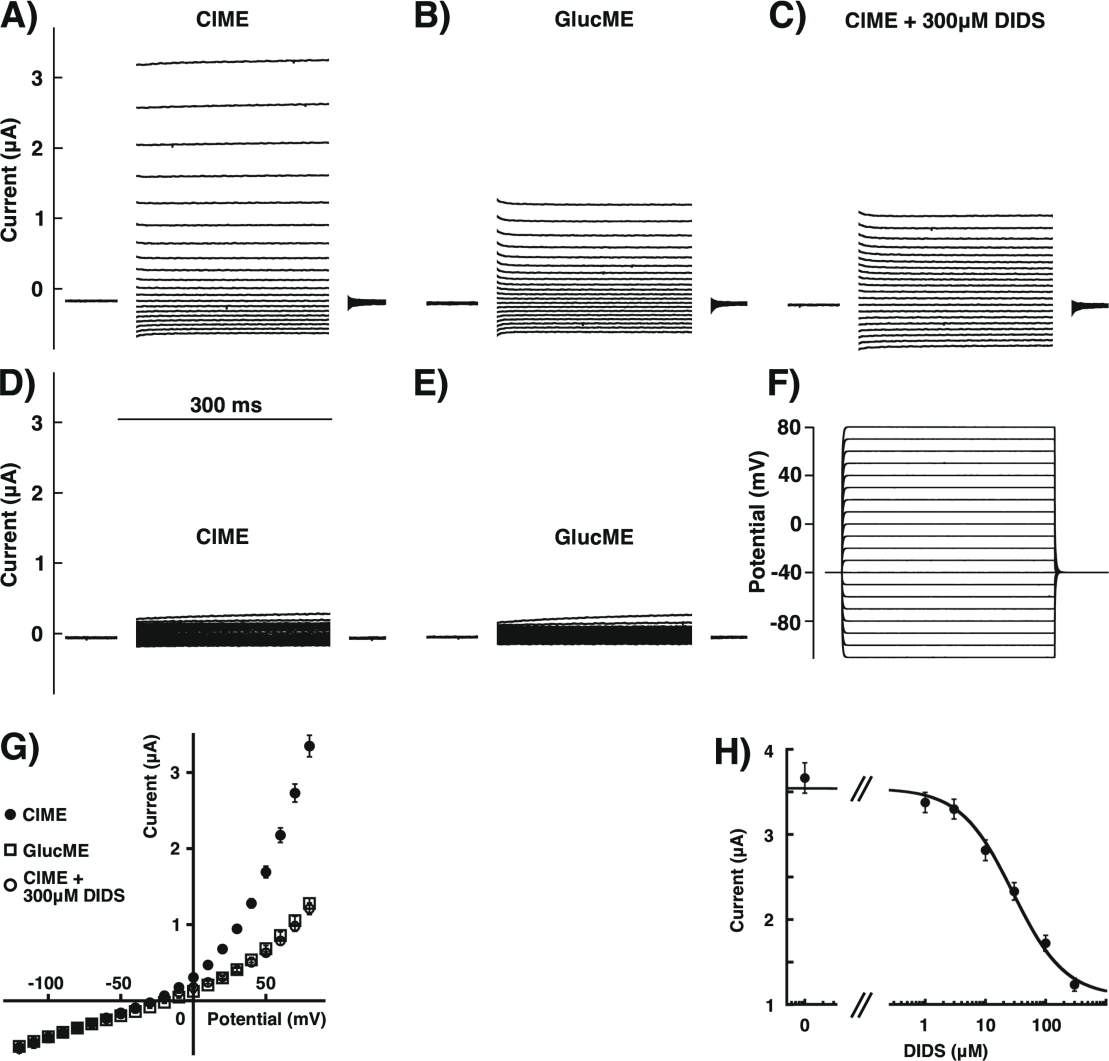
Electrophysiological characterization of TbVCL2 expressed in *Xenopus laevis* oocytes. The 2-electrode voltage-clamp was used to study currents in oocytes injected with cRNA coding for TbVCL2 or water. All recordings were done by applying the voltage-step protocol depicted in (F). The holding potential was -40 mV. With a frequency of 1 Hz, voltage-steps of 300 ms duration were applied from -100 mV to +80 mV in 10 mV intervals. A representative example of current traces recorded from an oocyte expressing TbVCL2 in media (A) with chloride as major anion (ClME), (B) with gluconate as major anion (GlucME) and (C) inhibition of chloride currents by 300 μM DIDS. For comparison, currents recorded from water-injected control oocytes in media with chloride as major anion (ClME) and gluconate as major anion (GlucME) are shown in (D) and (E). (G) averaged I/V-relationships obtained using the voltage-step protocol shown in panel F from TbVCL2-expressing oocytes (mean ± SEM, n = 19–29). Substitution of chloride by gluconate (GlucME, open squares) leads to a substantial reduction of the outward currents observed during pulses to positive potentials compared to medium containing chloride as major anion (ClME, closed circles). Addition of 300 μM DIDS (open circles) to the medium containing chloride as major anion led to a similar reduction of the outward currents. (H) Concentration-dependent inhibition of the current observed at +80 mV of TbVCL2-expressing oocytes in ClME with increasing concentrations of DIDS (mean ± SEM, n = 6). The inhibition was fitted with an IC_50_ of 30 ± 3 μM.

To characterize TbVCL2, voltage steps in intervals of 10 mV, from -120 mV to +80 mV, were applied from a holding potential of -40 mV (Fig 3F). Perfusion of cRNA-injected oocytes with chloride medium, containing chloride as the major anion, resulted in large outward rectifying currents (Fig 3A). We did not observe any time-dependent variation of the current during the 300 ms pulses. This is in contrast to the slowly inactivating endogenous anion conductance that seems to be activated in oocytes injected with mRNA coding for CLC-6 or a putative soluble protein [39]. Replacing Cl^-^ with gluconate led to a significant decrease in current amplitudes during pulses to positive potentials (Fig 3B). A similar effect was observed when 300 μM 4,4-diisothiocyanostilbene-2,2-disulphonate (DIDS) was added to the chloride medium (Fig 3C). Inhibition by DIDS was fully reversible. The fact that at negative potentials inward currents were similar in the presence of DIDS as in chloride and gluconate medium indicates little inhibition in this voltage range. Representative current traces obtained in experiments with oocytes injected with water are also shown (Fig 3D and 3E). Experiments were performed in chloride and gluconate medium, respectively. It should be noted that the membrane of oocytes injected with RNA coding for TbVCL2 displayed a higher conductance than oocytes injected with water. It is not clear whether this extra conductance reflects non-specific damage to the oocytes or an extra conductance induced by expression of TbVCL2.

Fig 3G shows current-voltage relationships of TbVCL2 currents recorded in chloride and gluconate medium, and chloride medium supplied with 300 μM DIDS. While inward currents at negative potentials were small, outward currents at positive potentials were larger than expected from the respective chloride concentrations inside (about 33 mM, as estimated from the reversal potential of currents mediated by chloride-selective GABA_A_ receptors expressed in oocytes) and outside (95 mM) the oocytes, indicating outward rectification. Experiments with TbVCL1 and TbVCL3 gave comparable results (S4 Fig). As mentioned above, DIDS acted as blocker of the current. Inhibition of TbVCL2 at +80 mV was fitted with an IC_50_ of 30 ± 3 μM (SEM, n = 6) (Fig 3H). TbVCL1 (n = 2) and TbVCL3 (n = 5) (S5 Fig) showed a similar sensitivity to DIDS.

**Fig 4:**
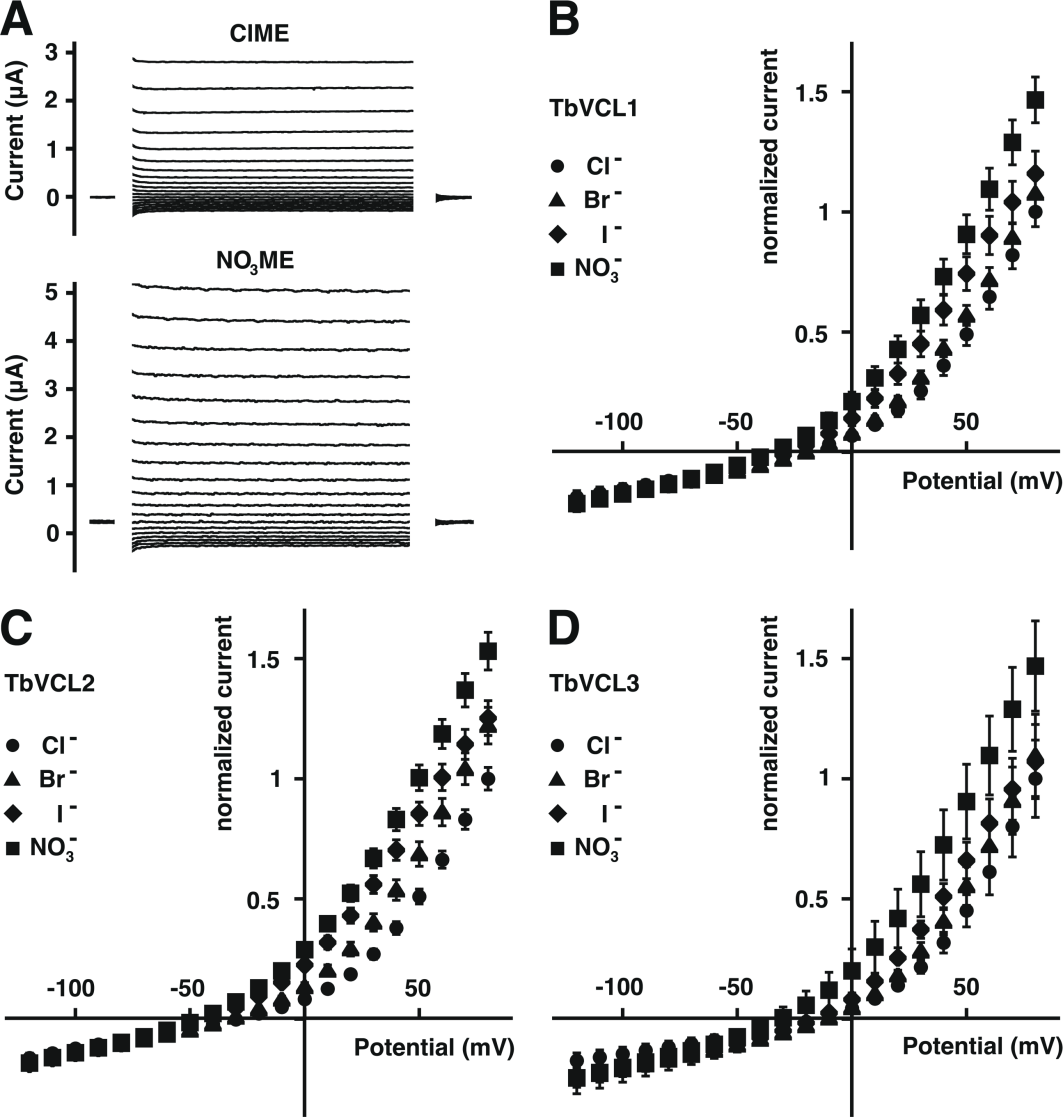
Determination of the relative anion selectivity of TbVCL1, TbVCL2 and TbVCL3. (A) Representative current traces of an oocyte expressing TbVCL2 in chloride medium (ClME; upper panel) and nitrate medium (NO_3_ME; lower panel). Currents of water-injected control oocytes recorded in the different media were averaged and then subtracted from the values obtained with cRNA-injected oocytes. These corrected currents were then normalized to the averaged current amplitude observed at +80 mV in chloride medium (2.65 ± 0.16 μA for TbVCL1; 2.74 ± 0.13 μA for TbVCL2 and 2.20 ± 0.35 μA for TbVCL3). The current-voltage relationships recorded from TbVCL1-, TbVCL2- and TbVCL3-expressing oocytes are shown in (B), (C) and (D), respectively (all values are mean ± SEM, n = 6–9 for TbVCL1; mean ± SEM, n = 6–10 for TbVCL2 and mean ± SD, n = 3 for TbVCL3). The major anion in the tested media are chloride (circles), bromide (triangles), iodide (diamonds) and nitrate (squares). For detailed media composition see section materials and methods.

In addition, we investigated the anion selectivity of the currents. Fig 4A illustrates original current traces recorded in Cl^-^ and NO_3_^-^ medium from an oocyte expressing TbVCL2. Fig 4B, 4C and 4D show averaged current voltage relationships of TbVCL1, TbVCL2 and TbVCL3, respectively, recorded in media with Cl^-^, Br^-^, I^-^ and NO_3_^-^ as major anion. For all three transport proteins, the anion selectivity was NO_3_^-^ > I^-^ > Br^-^ > Cl^-^.

We found that the current amplitudes at +80 mV elicited by the individual transport proteins were independent of the pH in the range of 5.4–8.4 (S6 Fig).

### Localization of TbVCL1, TbVCL2 and TbVCL3 in *T*. *brucei*

**Fig 5:**
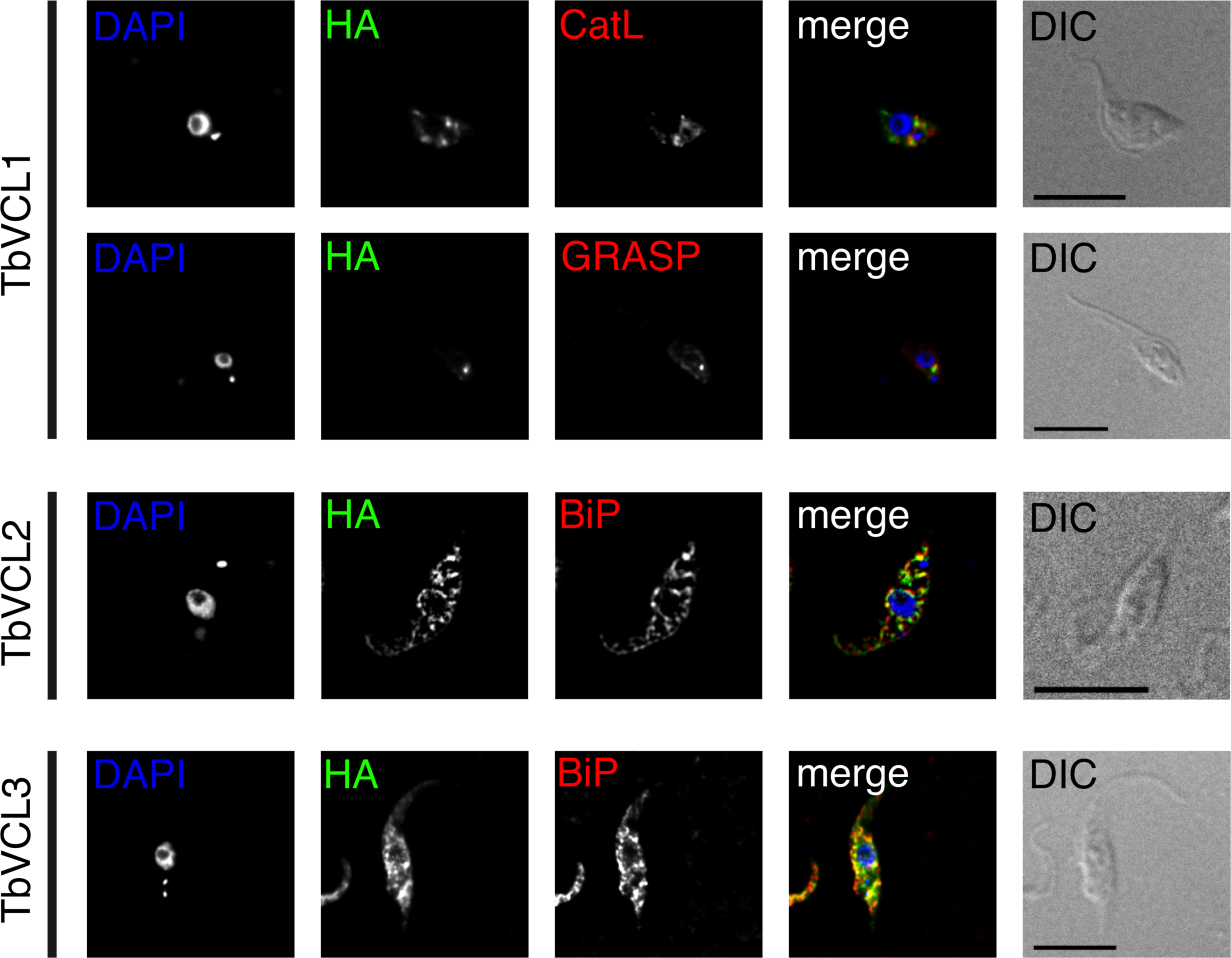
Localization of TbVCL1, TbVCL2 and TbVCL3 in *T*. *brucei* procyclic forms. C-terminally HA-tagged TbVCL1 and N-terminally tagged TbVCL2 and TbVCL3 were co-localized with markers for organelles: CatL, lysosome; GRASP, Golgi; BiP, endoplasmic reticulum. TbVCL1 shows a unique localization within the group of TbVCLs, localizing to a few puncta in proximity of the nucleus. For TbVCL2 and TbVCL3, co-staining with the endoplasmic reticulum marker is shown. Cells were counterstained with DAPI, shown in blue, visualizing the nuclear and kinetoplast DNA. DIC, differential interference contrast. Scale bars indicate 10 μm.

To determine the localization of the three anion transport proteins in *T*. *brucei* procyclic forms, we attempted to stably express tetracycline-inducible C- and N-terminal hemagglutinin (HA)-tagged versions of TbVCL1, TbVCL2 and TbVCL3. TbVCL1 only showed fluorescence with the C-terminal tag while TbVCL2 and TbVCL3 showed fluorescence only in the N-terminally tagged version. Expression was confirmed by Western blotting (S7 Fig). Cell lines showing expression were analyzed 24 h post-induction by immunofluorescence microscopy (Fig 5). TbVCL1 showed a unique localization within the group of TbVCLs, localizing to a few puncta in proximity to the nucleus (Fig 5). Quantification of the number of puncta per cell in a total of 435 parasites in two biological replicates (n_1_ = 256, n_2_ = 179) revealed that 53% of parasites contained one major punctum, with the remainder containing either no (6%), two (29%) or more (12%) puncta of similar intensity. The puncta showed no co-localization with lysosomes (CatL) or the Golgi (TbGRASP), however in most cases they localized close to TbGRASP (S8 Fig). For cells with one kinetoplast and one nucleus (1K1N) and containing one TbVCL1 punctum, we determined the distance between the Golgi signal and the HA signal to be 0.5 ± 0.1 μm (mean ± SD, n = 17). In 1K1N cells, we counted an average of 1.4 ± 0.8 puncta (n = 393), in 2K1N cells 2.2 ±1.0 (n = 27) and in 2K2N cells 3.0 ± 1.6 (n = 15).

For TbVCL2 and TbVCL3, co-staining with the endoplasmic reticulum marker BiP showed good co-localization (Fig 5). Little co-localization was observed using mitotracker as mitochondrial marker. A statistical evaluation of both experiments is shown in S1 Table.

### RNA-interference (RNAi)-mediated knock-down of TbVCL1, TbVCL2 and TbVCL3 in procyclic form parasites

**Fig 6:**
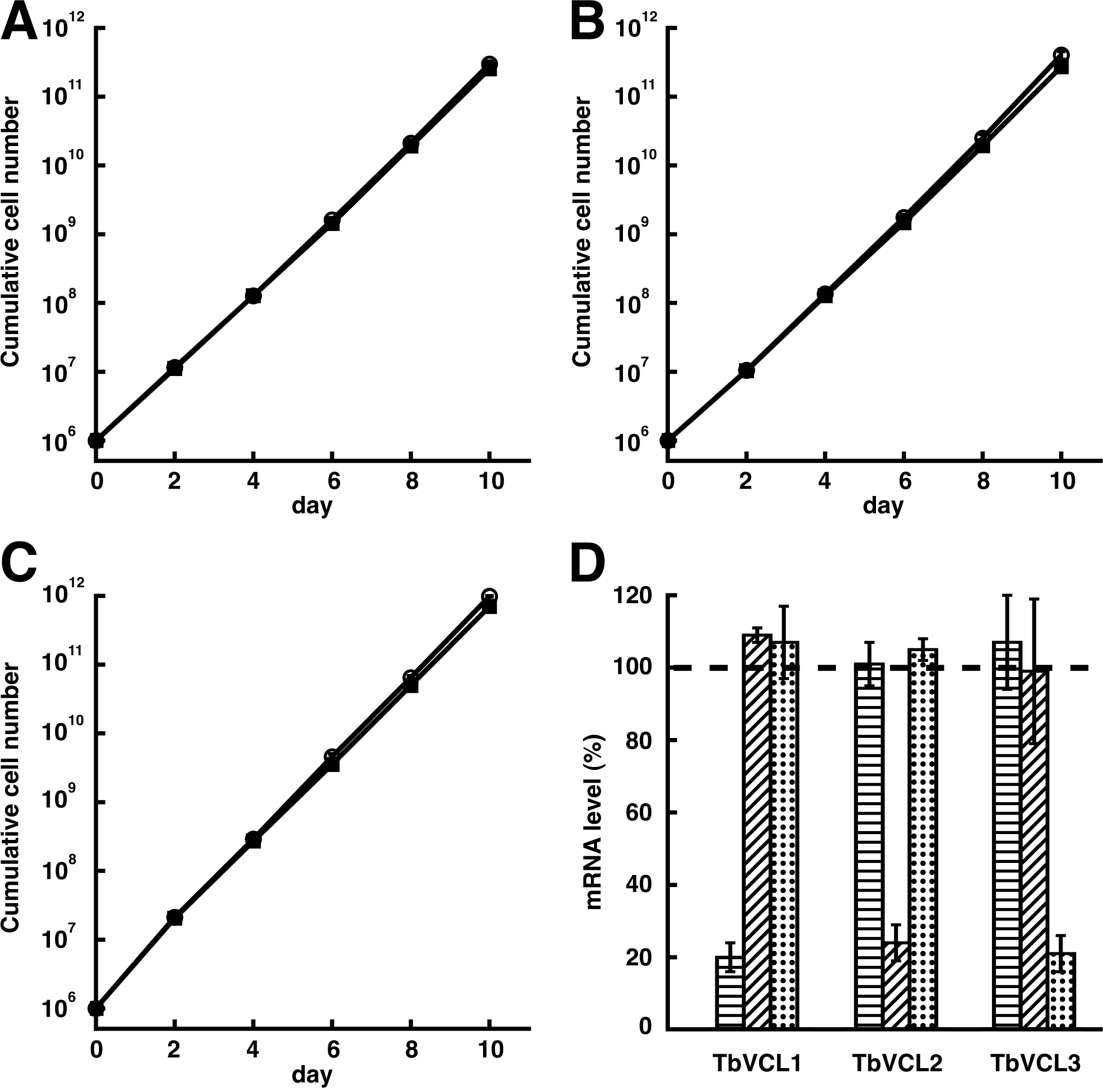
Down-regulation of TbVCL1, TbVCL2 and TbVCL3 by tetracycline-induced RNAi and its effect on parasite growth. Growth of procyclic parasites in absence (open circles) and presence (squares) of 1μg / mL tetracycline was monitored for 10 days. Induction of RNAi against TbVCL1 (A), TbVCL2 (B) or TbVCL3 (C) showed no growth retardation. All data points represent means from three independent experiments. (D) mRNA levels of TbVCL1, TbVCL2 and TbVCL3 were determined by qRT-PCR 48 h post induction. All values were normalized to mRNA level of the respective RNAi-target in non-induced cells (n = 3; mean ± SD). Horizontal striped bars represent the results using TbVCL1-specific primers, diagonal striped bars represent the results using TbVCL2-specific primers and dotted bars represent the results using TbVCL3-specific primers.

Essentiality of TbVCL1, TbVCL2 and TbVCL3 expression for growth of *T. brucei* procyclic forms in culture was investigated using tetracycline-inducible RNAi. The results show that addition of tetracycline to the culture medium had no effect on parasite proliferation (Fig 6A, 6B and 6C). To verify the efficiency of the RNAi constructs, we isolated total RNA 48 h after induction and determined the changes in mRNA levels by qPCR in three independent experiments. We found a down-regulation of 80 ± 4%, 76 ± 6% and 79 ± 5% (means ± SDs) for TbVCL1, TbVCL2 and TbVCL3, respectively (Fig 6D). Down-regulation of one of the proteins did not significantly affect mRNA abundance of the other two proteins in all three cases (Fig 6D).

Because of the overlapping localization of TbVCL2 and TbVCL3 (see above), we decided to downregulate the expression of these two proteins in the same cell line. After transfecting the TbVCL2 RNAi cell line with a second RNAi construct targeting TbVCL3, we obtained a downregulation of 74% and 62% for TbVCL2 and TbVCL3, respectively, in one biological duplicate, and 73% and 58% in the other one. Despite the reduction in TbVCL2 and TbVCL2 mRNA levels, no difference in growth was observed between induced and non-induced cells over seven days (S9 Fig).

## Discussion

We describe the identification and localization of three different anion transport proteins in *T*. *brucei*, named TbVCL1, TbVCL2 and TbVCL3. The three proteins share only low amino acid residue identity of < 27% amongst each other. They are also quite distant from the closest human homologs CLC-6 and CLC-7 with an identity of < 27%. CLC-6 and CLC-7 have been shown to catalyze electrogenic Cl^-^/H^+^-exchange [40,41] and are localized intracellularly. CLC-7 is associated with the β-subunit Ostm1 [12]. We did not find any candidate gene in *T*. *brucei* coding for a putative β-subunit of chloride transport proteins. In the following we compare the TbVCLs with human CLC-6 and CLC-7.

TbVCL1, TbVCL2 and TbVCL3 induced chloride currents upon expression in *Xenopus* oocytes. As mentioned above, we can not exclude that these proteins form dimers with endogenous CLC like proteins. In all three cases only a fraction of injected oocytes showed extra chloride currents over the plasma membrane. In case of human CLC-6 efficient re-routing to the oocyte plasma membrane required N-terminal attachment of GFP [40] and in case of CLC-7/Ostm1 mutations in the signal sequence of CLC-7 [41] were required. Currents induced by TbVCL2 showed little time dependence and were outwardly rectifying. Currents mediated by human CLC-6 showed little time and voltage dependence [40], while CLC-7/Ostm1 showed both [41]. We failed to demonstrate pH-dependence while for CLC-6 and CLC-7 such pH effects are well documented [40,41].

The anion selectivity of all three TbVCLs was NO_3_^-^ > I^-^ > Br^-^ > Cl^-^. Anion selectivity of TbVCL1, TbVCL2 and TbVCL3 is different from CLC-6 with NO_3_^-^ > Cl^-^ > I^-^ [40] and CLC-7/Ostm1 with Cl^-^ > Br^-^ > NO_3_^-^ > I^-^ [41].

Like many Cl^-^ conducting proteins, the currents induced by TbVCL1, TbVCL2 and TbVCL3 were reversibly inhibited by DIDS. Other examples of the CLC family that show reversible inhibition by DIDS include CLC-Ka, CLC-2 and CLC-ec1 [42–44]. The IC_50_ value for TbVCL2 was about 30 μM. DIDS sensitivity of the *Arabidopsis* AtClCa transporter has not been reported.

While all three TbVCLs induced currents in oocytes, only TbVCL1 was able to complement the phenotype of the *S*. *cerevisiae gef1∆* mutant. It is interesting to note that CLC-6, but not CLC-7 is able to rescue the growth phenotype of this mutant [45]. Of the 7 *Arabidopsis* CLCs, only AtClCd and AtClCf were able to complement the *gef1* mutant phenotype [46]. Consistent with these results and similar to the Gef1 protein, AtClCd and AtClCf were so far the only members from *Arabidopsis* localized in the Golgi. In contrast, at least two of the *Arabidopsis* CLC transporters, AtClCa and AtClCe, that failed to complement the *gef1∆* mutant phenotype were localized in chloroplast membranes and at the tonoplast, respectively [47].

Localization of the anion transport proteins was investigated in *T*. *brucei* procyclic forms. TbVCL1 showed a different intracellular localization than TbVCL2 and TbVCL3. The few puncta detected in proximity of the nucleus did not co-localize with markers for lysosomes or Golgi. We speculate that nonetheless, TbVCL1 might be associated with the Golgi apparatus, mainly because of the adjacent localization of the HA-TbVCL1 signal to the Golgi marker TbGRASP. The average number of HA-TbVCL1 puncta per cell (1.4 and 2.5, respectively) correlated with the number of kinetoplasts (1 and 2, respectively), the correlation coefficient, however, was low (r = 0.35). The correlation coefficients of the two indicators, HA-TbVCL1 and Golgi apparatus, showed only weak correlation, too (r = 0.36). Interestingly, TbVCL1 compensated for loss of a *S*. *cerevisiae* chloride channel that locates to the late Golgi and the prevacuole in this organism [48]. In contrast, the other two transporters, TbVCL2 and TbVCL3, showed partial co-localization with the endoplasmic reticulum marker BiP. This result should be taken with care as localization to the endoplasmic reticulum might be an effect of overexpression and/or maybe due to lack of a binding partner. In a recent study, TbVCL2 was N-terminally GFP-tagged in procyclic form parasites. This localization experiment resulted in punctate staining which was, despite of the lack of a marker, interpreted as endocytic localization [49]. The reason for the different localization is not clear.

In summary, three putatively independent anion transport proteins of the CLC family have been identified in *T*. *brucei*. Yeast complementation experiments indicate that at least TbVCL1 works as a genuine chloride transporter.

## Supporting information

S1 Fig Robustness of the tree. Robustness of the tree depicted in Fig 1 was tested by applying the bootstrap method with 1000 replications. The resulting values are shown next to the corresponding nodes.
(PDF)

S2 Fig Alignment of TbVCL1, TbVCL2 and TbVCL3 to known CLC proteins. The three *T*. *brucei* proteins were aligned to known CLC proteins by MUSCLE. Key residues for the permeation pathway are highlighted following their numbering in *E*. *coli*; S107, Y445, and E148 –also known as gating glutamate or E_ext_. In addition, E203 –also known as the proton glutamate or E_int_−is depicted, it is an additional key residue for chloride transport in ClC-ec1. Residue background coloring represents amount of identity ranging from dark blue (0%) to bright red (100%), summarized below the alignment.
(PDF)

S3 Fig TbVCLs transcript level in TbVCL-expressing *S*. *cerevisiae*. The mRNA abundance in TbVCL1, TbVCL2 and TbVCL3-expressing yeast, respectively, was quantified by qRT-PCR and normalized to reference genes ALG9 and TAF10, respectively. TbVCL mRNA was not detected in yeast cells transformed with empty vector. The values are mean ± SD of three technical replicates.
(PDF)

S4 Fig I/V curves of TbVCL1- and TbVCL3-expressing oocytes. The current-voltage relationships were determined in medium containing chloride as major anion (ClME, closed circles), in medium containing gluconate as major anion (GlucME, open squares) and chloride medium supplemented with 300 μM DIDS (open circles). Substitution of chloride by gluconate leads to a substantial reduction of the outward currents observed during pulses to positive potentials compared to medium containing chloride as major anion. Averaged I/V-relationships obtained from TbVCL1-expressing oocytes (mean ± SEM, n = 11–16) and TbVCL3-expressing oocytes (mean ± SEM, n = 4–9) are shown in panel (A) and (B), respectively.
(PDF)

S5 Fig Sensitivity to DIDS of TbVCL3-expressing oocytes. Concentration-dependent inhibition of the current observed at +80 mV of TbVCL3-expressing oocytes in chloride medium with increasing concentrations of DIDS (mean ± SEM, n = 5). The inhibition was fitted with an IC_50_ of 56 ± 18 μM.
(PDF)

S6 Fig Current amplitudes observed in TbVCL-expressing oocytes in the pH-range from 5.4 to 8.4. All measurements were performed with chloride medium. Measured steady-state currents at a potential of +80 mV were normalized to the value observed at pH 7.4 (mean ± SD, n = 3, n = 5 and n = 3 for TbVCL1, TbVCL2 and TbVCL3, respectively). We did not find an influence of the pH on the current amplitudes in the measured range.
(PDF)

S7 Fig Western blot of HA-tagged versions of TbVCL1, TbVCL2 and TbVCL3. Induced (+) and non-induced (-) *T*. *brucei* cell lines generated for localization experiments were analyzed by Western blot to confirm overexpression of the tagged TbVCLs. About 5x10^6^ cells of each clone were subjected to 8% SDS-polyacrylamide gel electrophoresis, transferred to nitrocellulose membranes, and probed with mouse anti-HA antibody. Expression of the tagged TbVCLs could be confirmed (expected size: 103 kDa for TbVCL1, 109 kDa for TbVCL2 and 102 kDa for TbVCL3; upper bands). As loading control the membranes were reprobed with rabbit anti-Bip antibody (expected size: 75 kDa; lower bands). Relative position of the protein-ladder is shown on the left side.
(PDF)

S8 Fig Close apposition of TbVCL1 to the Golgi apparatus. Ten representative procyclic *T*. *brucei* cells are shown as immunofluorescence image and right below the same transmitted light microscopy pictures obtained by differential interference contrast. C-terminally HA-tagged TbVCL1 is shown in green, the Golgi marker TbGRASP in red and the nuclear and kinetoplast DNA stain DAPI in blue. Scale bars indicate 10 μm. TbVCL1 localizes always close to the Golgi apparatus.
(PDF)

S9 Fig Effect on parasite growth after down-regulation of TbVCL2 and TbVCL3 by tetracycline-induced RNAi in the same cell line. Cumulative growth of procyclic parasites in absence (open circles) and presence (squares) of 1μg / mL tetracycline was monitored for 7 days. Two biological duplicates are shown (A and B). Induction of RNAi against both TbVCL2 and TbVCL3 showed no growth retardation under standard culture conditions. All data points represent means from two independent experiments.
(PDF)

S1 Table Statistical evaluation of TbVCL2 and TbVCL3 co-localization with the ER-marker BiP and Mitotracker, respectively. (PDF)
