# Supplementary material for: Identification and characterization of the three members of the CLC family of anion transport proteins in Trypanosoma brucei
Source: PLoS One. 2017 Dec 15;12(12):e0188219. doi: 10.1371/journal.pone.0188219 (PMC5731698; doi:10.1371/journal.pone.0188219)
Supplement: S1 Fig — Robustness of the tree depicted in Fig 1 was tested by applying the bootstrap method with 1000 replications. The resulting values are shown next to the corresponding nodes. (PDF) [file pone.0188219.s001.pdf]

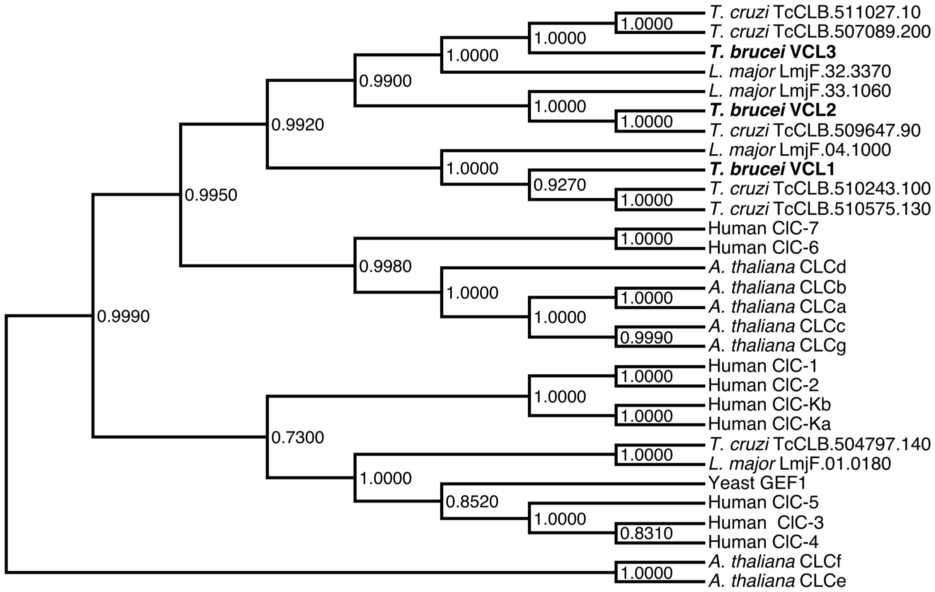

S1 Fig. Robustness of the tree. Robustness of the tree depicted in Fig. 1 was tested by applying the bootstrap method with 1000 replications. The resulting values are shown next to the corresponding nodes.
