# Supplementary material for: Identification and characterization of the three members of the CLC family of anion transport proteins in Trypanosoma brucei
Source: PLoS One. 2017 Dec 15;12(12):e0188219. doi: 10.1371/journal.pone.0188219 (PMC5731698; doi:10.1371/journal.pone.0188219)
Supplement: S2 Fig — The three T. brucei proteins were aligned to known CLC proteins by MUSCLE. Key residues for the permeation pathway are highlighted following their numbering in E. coli; S107, Y445, and E148 –also known as gating glutamate or Eext. In addition, E203 –also known as the proton glutamate or Eint−is depicted, it is an additional key residue for chloride transport in ClC-ec1. Residue background coloring represents amount of identity ranging from dark blue (0%) to bright red (100%), summarized below the alignment. (PDF) [file pone.0188219.s002.pdf]

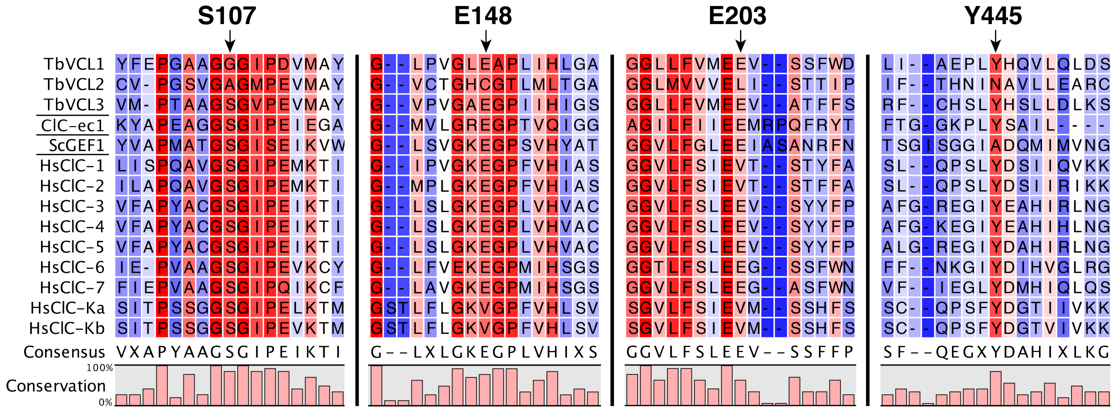

S2 Fig. Alignment of TbVCL1, TbVCL2 and TbVCL3 to known CLC proteins. The three *T. brucei* proteins were aligned to known CLC proteins by MUSCLE. Key residues for the permeation pathway are highlighted following their numbering in *E. coli*; S107, Y445, and E148 – also known as gating glutamate or E_{ext}. In addition, E203 – also known as the proton glutamate or E_{int} – is depicted, it is an additional key residue for chloride transport in ClC-ec1. Residue background coloring represents amount of identity ranging from dark blue (0%) to bright red (100%), summarized below the alignment.
