# Supplementary material for: Identification and characterization of the three members of the CLC family of anion transport proteins in Trypanosoma brucei
Source: PLoS One. 2017 Dec 15;12(12):e0188219. doi: 10.1371/journal.pone.0188219 (PMC5731698; doi:10.1371/journal.pone.0188219)
Supplement: S3 Fig — The mRNA abundance in TbVCL1, TbVCL2 and TbVCL3-expressing yeast, respectively, was quantified by qRT-PCR and normalized to reference genes ALG9 and TAF10, respectively. TbVCL mRNA was not detected in yeast cells transformed with empty vector. The values are mean ± SD of three technical replicates. (PDF) [file pone.0188219.s003.pdf]

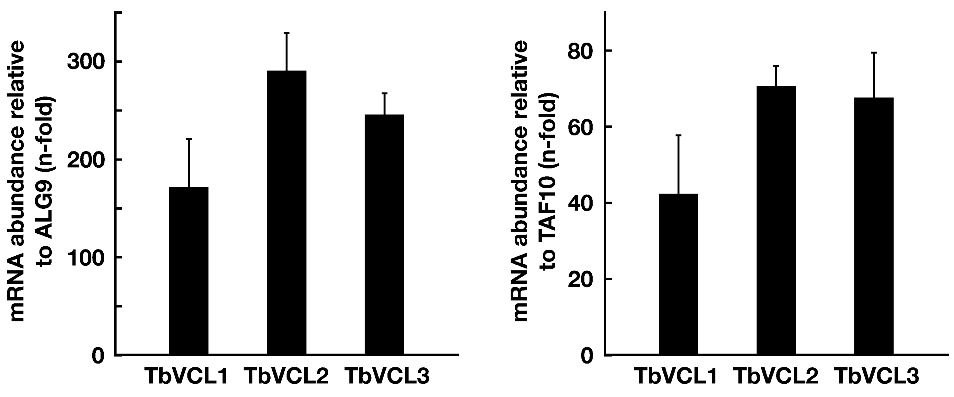

S3 Fig. TbVCLs transcript level in TbVCL-expressing *S. cerevisiae*. The mRNA abundance in TbVCL1, TbVCL2 and TbVCL3-expressing yeast, respectively, was quantified by qRT-PCR and normalized to reference genes ALG9 and TAF10, respectively. TbVCL mRNA was not detected in yeast cells transformed with empty vector. The values are mean ± SD of three technical replicates.
