# Supplementary material for: Identification and characterization of the three members of the CLC family of anion transport proteins in Trypanosoma brucei
Source: PLoS One. 2017 Dec 15;12(12):e0188219. doi: 10.1371/journal.pone.0188219 (PMC5731698; doi:10.1371/journal.pone.0188219)
Supplement: S4 Fig — The current-voltage relationships were determined in medium containing chloride as major anion (ClME, closed circles), in medium containing gluconate as major anion (GlucME, open squares) and chloride medium supplemented with 300 μM DIDS (open circles). Substitution of chloride by gluconate leads to a substantial reduction of the outward currents observed during pulses to positive potentials compared to medium containing chloride as major anion. Averaged I/V-relationships obtained from TbVCL1-expressing oocytes (mean ± SEM, n = 11–16) and TbVCL3-expressing oocytes (mean ± SEM, n = 4–9) are shown in panel (A) and (B), respectively. (PDF) [file pone.0188219.s004.pdf]

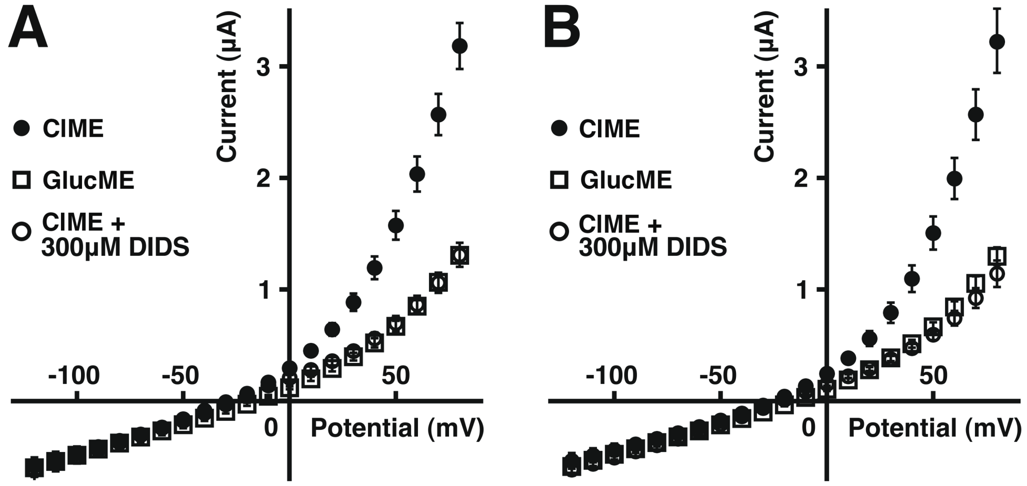

S4 Fig. I/V curves of TbVCL1- and TbVCL3-expressing oocytes. The current-voltage relationships were determined in medium containing chloride as major anion (ClME, closed circles), in medium containing gluconate as major anion (GlucME, open squares) and chloride medium supplemented with 300 µM DIDS (open circles). Substitution of chloride by gluconate leads to a substantial reduction of the outward currents observed during pulses to positive potentials compared to medium containing chloride as major anion. Averaged I/V-relationships obtained from TbVCL1-expressing oocytes (mean ± SEM, n = 11-16) and TbVCL3-expressing oocytes (mean ± SEM, n = 4-9) are shown in panel (A) and (B), respectively.
