# Supplementary material for: Identification and characterization of the three members of the CLC family of anion transport proteins in Trypanosoma brucei
Source: PLoS One. 2017 Dec 15;12(12):e0188219. doi: 10.1371/journal.pone.0188219 (PMC5731698; doi:10.1371/journal.pone.0188219)
Supplement: S5 Fig — Concentration-dependent inhibition of the current observed at +80 mV of TbVCL3-expressing oocytes in chloride medium with increasing concentrations of DIDS (mean ± SEM, n = 5). The inhibition was fitted with an IC50 of 56 ± 18 μM. (PDF) [file pone.0188219.s005.pdf]

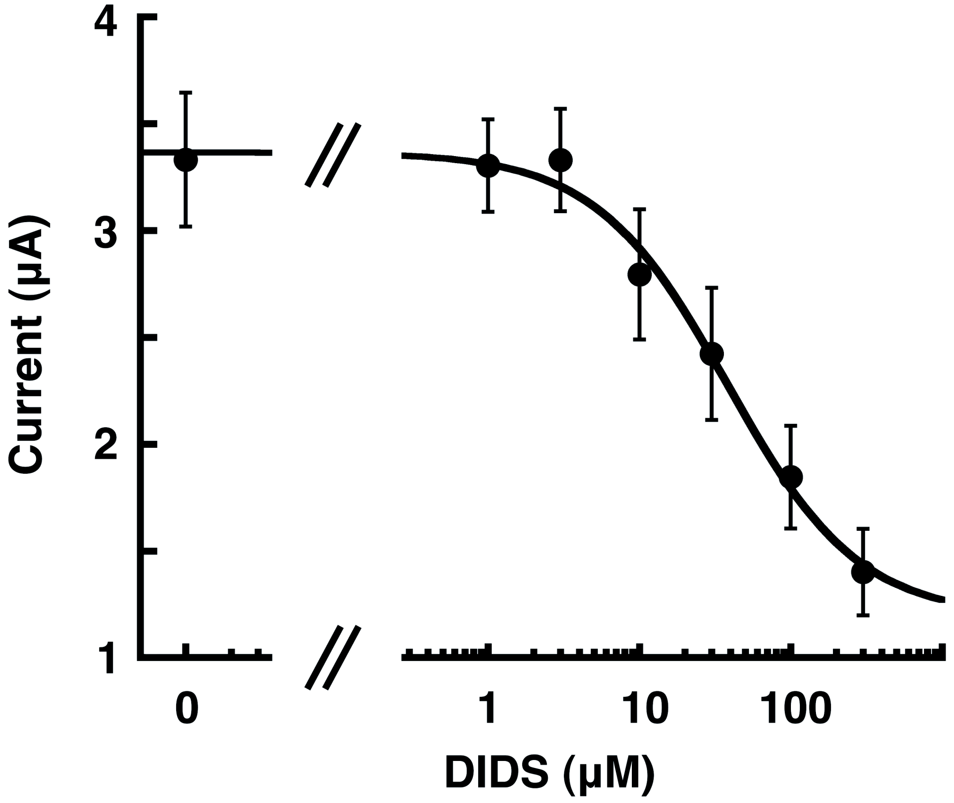

S5 Fig. Sensitivity to DIDS of TbVCL3-expressing oocytes. Concentration-dependent inhibition of the current observed at +80 mV of TbVCL3-expressing oocytes in chloride medium with increasing concentrations of DIDS (mean ± SEM, n = 5). The inhibition was fitted with an IC_{50} of 56 ± 18 µM.
