# Supplementary material for: Identification and characterization of the three members of the CLC family of anion transport proteins in Trypanosoma brucei
Source: PLoS One. 2017 Dec 15;12(12):e0188219. doi: 10.1371/journal.pone.0188219 (PMC5731698; doi:10.1371/journal.pone.0188219)
Supplement: S6 Fig — All measurements were performed with chloride medium. Measured steady-state currents at a potential of +80 mV were normalized to the value observed at pH 7.4 (mean ± SD, n = 3, n = 5 and n = 3 for TbVCL1, TbVCL2 and TbVCL3, respectively). We did not find an influence of the pH on the current amplitudes in the measured range. (PDF) [file pone.0188219.s006.pdf]

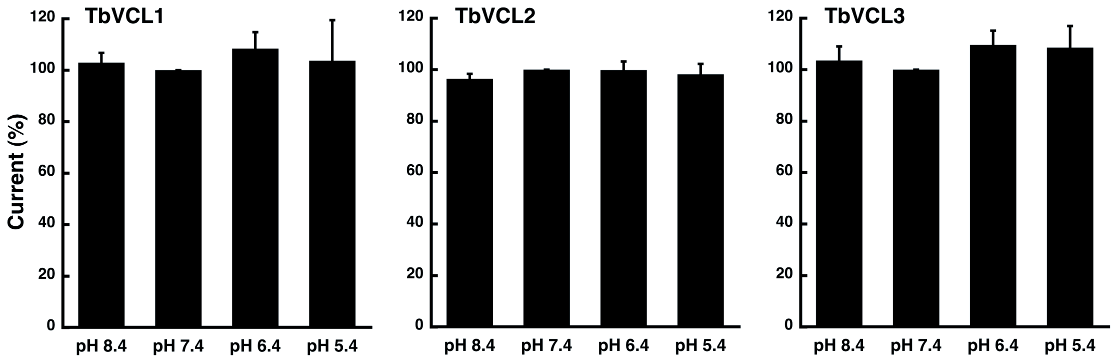

S6 Fig. Current amplitudes observed in TbVCL-expressing oocytes in the pH-range from 5.4 to 8.4. All measurements were performed with chloride medium. Measured steady-state currents at a potential of +80 mV were normalized to the value observed at pH 7.4 (mean ± SD, n = 3, n = 5 and n = 3 for TbVCL1, TbVCL2 and TbVCL3, respectively). We did not find an influence of the pH on the current amplitudes in the measured range.
