# Supplementary material for: Identification and characterization of the three members of the CLC family of anion transport proteins in Trypanosoma brucei
Source: PLoS One. 2017 Dec 15;12(12):e0188219. doi: 10.1371/journal.pone.0188219 (PMC5731698; doi:10.1371/journal.pone.0188219)
Supplement: S7 Fig — Induced (+) and non-induced (-) T. brucei cell lines generated for localization experiments were analyzed by Western blot to confirm overexpression of the tagged TbVCLs. About 5x10^6^ cells of each clone were subjected to 8% SDS-polyacrylamide gel electrophoresis, transferred to nitrocellulose membranes, and probed with mouse anti-HA antibody. Expression of the tagged TbVCLs could be confirmed (expected size: 103 kDa for TbVCL1, 109 kDa for TbVCL2 and 102 kDa for TbVCL3; upper bands). As loading control the membranes were reprobed with rabbit anti-Bip antibody (expected size: 75 kDa; lower bands). Relative position of the protein-ladder is shown on the left side. (PDF) [file pone.0188219.s007.pdf]

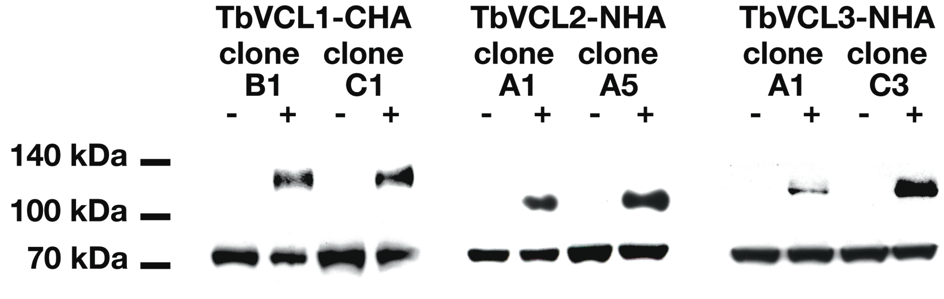

S7 Fig. Western blot of HA-tagged versions of TbVCL1, TbVCL2 and TbVCL3. Induced (+) and non-induced (-) *T. brucei* cell lines generated for localization experiments were analyzed by Western blot to confirm overexpression of the tagged TbVCLs. About $5x10^6$ cells of each clone were subjected to 8% SDS-polyacrylamide gel electrophoresis, transferred to nitrocellulose membranes, and probed with mouse anti-HA antibody. Expression of the tagged TbVCLs could be confirmed (expected size: 103 kDa for TbVCL1, 109 kDa for TbVCL2 and 102 kDa for TbVCL3; upper bands). As loading control the membranes were reprobed with rabbit anti-Bip antibody (expected size: 75 kDa; lower bands). Relative position of the protein-ladder is shown on the left side.
