# Supplementary material for: Identification and characterization of the three members of the CLC family of anion transport proteins in Trypanosoma brucei
Source: PLoS One. 2017 Dec 15;12(12):e0188219. doi: 10.1371/journal.pone.0188219 (PMC5731698; doi:10.1371/journal.pone.0188219)
Supplement: S8 Fig — Ten representative procyclic T. brucei cells are shown as immunofluorescence image and right below the same transmitted light microscopy pictures obtained by differential interference contrast. C-terminally HA-tagged TbVCL1 is shown in green, the Golgi marker TbGRASP in red and the nuclear and kinetoplast DNA stain DAPI in blue. Scale bars indicate 10 μm. TbVCL1 localizes always close to the Golgi apparatus. (PDF) [file pone.0188219.s008.pdf]

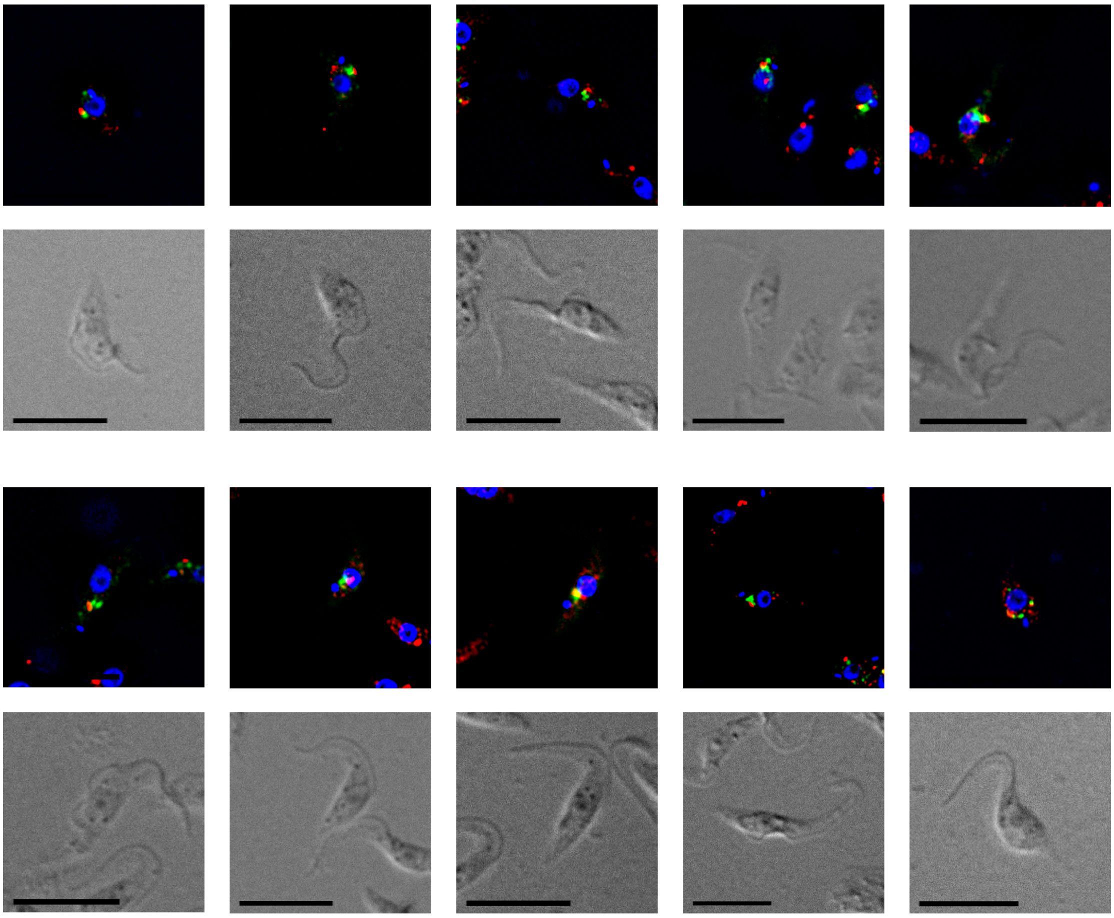

S8 Fig. Close apposition of TbVCL1 to the Golgi apparatus. Ten representative procyclic *T. brucei* cells are shown as immunofluorescence image and right below the same transmitted light microscopy pictures obtained by differential interference contrast. C-terminally HA-tagged TbVCL1 is shown in green, the Golgi marker TbGRASP in red and the nuclear and kinetoplast DNA stain DAPI in blue. Scale bars indicate 10 µm. TbVCL1 localizes always close to the Golgi apparatus.
