# Supplementary material for: Identification and characterization of the three members of the CLC family of anion transport proteins in Trypanosoma brucei
Source: PLoS One. 2017 Dec 15;12(12):e0188219. doi: 10.1371/journal.pone.0188219 (PMC5731698; doi:10.1371/journal.pone.0188219)
Supplement: S9 Fig — Cumulative growth of procyclic parasites in absence (open circles) and presence (squares) of 1μg / mL tetracycline was monitored for 7 days. Two biological duplicates are shown (A and B). Induction of RNAi against both TbVCL2 and TbVCL3 showed no growth retardation under standard culture conditions. All data points represent means from two independent experiments. (PDF) [file pone.0188219.s009.pdf]

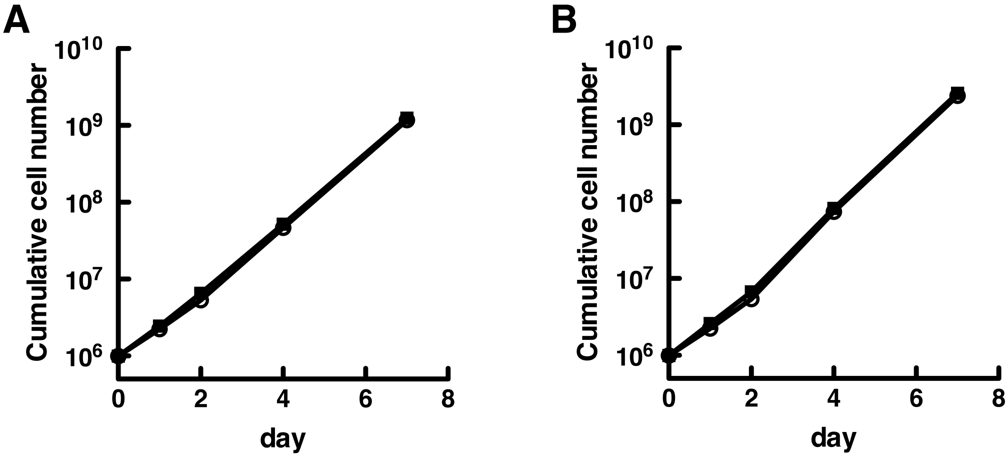

S9 Fig. Effect on parasite growth after down-regulation of TbVCL2 and TbVCL3 by tetracycline-induced RNAi in the same cell line. Cumulative growth of procyclic parasites in absence (open circles) and presence (squares) of 1µg / mL tetracycline was monitored for 7 days. Two biological duplicates are shown (A and B). Induction of RNAi against both TbVCL2 and TbVCL3 showed no growth retardation under standard culture conditions. All data points represent means from two independent experiments.
