# Supplementary material for: Identification and characterization of the three members of the CLC family of anion transport proteins in Trypanosoma brucei
Source: PLoS One. 2017 Dec 15;12(12):e0188219. doi: 10.1371/journal.pone.0188219 (PMC5731698; doi:10.1371/journal.pone.0188219)
Supplement: S1 Table — (PDF) [file pone.0188219.s010.pdf]

ER

	TbVCL2								TbVCL3							
	Clone A1				Clone A5				Clone A1				Clone C3			
	1K1N	2K1N	2K2N	total	1K1N	2K1N	2K2N	total	1K1N	2K1N	2K2N	total	1K1N	2K1N	2K2N	total
**Pearson's r, average**	0.64	0.64	0.62	0.64	0.64	0.66	0.67	0.64	0.58	0.57	0.61	0.58	0.68	0.66	0.68	0.68
**Standard deviation**	0.08	0.05	0.07	0.07	0.09	0.10	0.09	0.09	0.11	0.12	0.10	0.11	0.09	0.05	0.02	0.08
**Standard error**	0.01	0.02	0.03	0.01	0.01	0.03	0.04	0.01	0.02	0.05	0.04	0.01	0.02	0.02	0.01	0.01
**n**	43	8	5	56	45	9	4	58	49	7	7	63	27	5	3	35

Colocalization between ER-marker BiP and HA-tagged TbVCL2 and TbVCL3, respectively.

Mito

	TbVCL2								TbVCL3							
	Clone A1				Clone A5				Clone A1				Clone C3			
	1K1N	2K1N	2K2N	total	1K1N	2K1N	2K2N	total	1K1N	2K1N	2K2N	total	1K1N	2K1N	2K2N	total
**Pearson's r, average**	0.30	0.21	0.32	0.3	0.34	0.36	0.34	0.35	0.30	0.27	0.28	0.3	0.31	0.30	0.31	0.31
**Standard deviation**	0.09	0.03	0.02	0.09	0.09	0.11	0.08	0.09	0.09	0.08	0.10	0.09	0.09	0.07	0.08	0.08
**Standard error**	0.01	0.01	0.01	0.01	0.01	0.03	0.03	0.01	0.01	0.02	0.05	0.01	0.01	0.02	0.03	0.01
**n**	48	4	4	56	69	11	7	87	95	12	4	111	60	10	6	76

Colocalization between Mitotacker and HA-tagged TbVCL2 and TbVCL3, respectively.

S1 Table Statistical evaluation of TbVCL2 and TbVCL3 co-localization with the ER-marker BiP and Mitotracker, respectively.
